# Tuberculosis, an unusual source - a case report

**DOI:** 10.47626/1679-4435-2021-805

**Published:** 2021-12-30

**Authors:** Alexandra Lima Roque, Silvia Maria Pimenta, Rita Assis Ribeiro, Ana Isabel Correia, Teresa Martinho Valente, Elvira Rodriguez Perea, Juan Carlos Fonnegra

**Affiliations:** 1 Serviço de Saúde Ocupacional, Centro Hospitalar Lisboa Ocidental, Lisboa, Lisboa, Portugal.; 2 Serviço de Saúde Ocupacional, Centro Hospitalar Lisboa Norte, Lisboa, Lisboa, Portugal.

**Keywords:** tuberculosis, pulmonary, tuberculosis, bovine, occupational health services, tuberculose pulmonar, tuberculose bovina, serviços de saúde do trabalhador

## Abstract

Tuberculosis is an infectious disease caused by bacteria in the *Mycobacterium tuberculosis* complex. It can affect any organ, but the pulmonary form is the most common manifestation. Not only humans can be affected by tuberculosis, and animals are also commonly infected. This disease can be transmitted to humans usually by inhalation of aerosols or by ingestion of unpasteurized milk or dairy products. We report the case of a zoo worker. He reported an epidemiological context of contact with sea lions and dolphins, of which he takes care, with tuberculosis in the last 4 months. He sought permanent medical care for a 3-week history of cough, fever, sweating, and weight loss. Bronchial lavage was positive for acid-alcohol resistant bacilli. Lavage cultures were positive for the *M. tuberculosis* complex. The patient was referred for treatment with antituberculosis drugs. Tuberculosis is a major public health problem worldwide. In the occupational setting, tuberculosis remains a matter of great concern and attention, most often in the hospital environment or among health care professionals. However, the case reported here shows another, less usual occupational setting in which this type of contact can also occur. It is expected that the warning of this case can be used by occupational health teams, namely those who are responsible for periodic surveillance of workers’ health in the animal sector.

## Resumo

A tuberculose é uma doença infecciosa causada por bactérias do *Mycobacterium tuberculosis complex*. Pode atingir qualquer órgão, sendo a forma pulmonar a mais frequente. Não só os humanos podem ser atingidos pela tuberculose, sendo também muito comum a existência de infecção de animais. Essa patologia pode espalhar-se para os humanos, normalmente pela inalação de aerossóis ou pela ingestão de leite ou derivados não pasteurizados. Trata-se do caso de um trabalhador de zoológico, com contexto epidemiológico de contato com leões-marinhos com tuberculose nos últimos 4 meses. Recorreu a atendimento permanente por história de 3 semanas de tosse, febre, sudorese e perda ponderal. A pesquisa de bacilos resistentes a álcool-ácido no lavado brônquico revelou-se positiva. Quanto ao exame cultural do lavado, concluiu-se positivo para *M. tuberculosis complex*. Foi, então, realizado o devido encaminhamento, e o paciente iniciou tratamento com antibacilares. A tuberculose constitui um importante problema de saúde pública em nível global. No contexto ocupacional, mantém-se como um motivo de grande preocupação e atenção, mais frequentemente em meio hospitalar ou entre os profissionais de saúde. No entanto, este caso mostra outro contexto ocupacional, menos óbvio, em que este tipo de contato também pode acontecer. Espera-se que o alerta do presente caso possa ser utilizado pelas equipas de saúde ocupacional, nomeadamente as que realizam vigilância periódica a trabalhadores do setor animal.

## INTRODUCTION

Tuberculosis is an infectious disease caused by bacteria in the *Mycobacterium tuberculosis* complex. It can affect any organ, but the pulmonary form is the most common manifestation and also the one with major implications in terms of public health. An ill person with pulmonary tuberculosis releases bacilli through coughing and speaking, among others, which will later be inhaled by people with whom they have been in contact.^1^

Not only humans can be affected by tuberculosis, and animals are also commonly infected. Animal tuberculosis is a chronic disease in animals, which is naturally transmitted from animals to humans, thus assuming the definition of zoonosis.^2^ This disease can be transmitted to humans usually by inhalation of aerosols and/or droplets or by ingestion of unpasteurized milk or dairy products.^3^ In this case, the disease is caused by *M. bovis*, a Gram-positive, rod-shaped, aerobic, immobile, acid-resistant bacterium, without capsule or spore, very similar to the bacterium causing human tuberculosis.^2^

It is believed that tuberculosis first emerged in East Africa as the *M. tuberculosis* bacillus and later spread from infected humans to animals, giving rise to *M. bovis*, now known as bovine tuberculosis, with the sale of animals being responsible for transmitting the disease.^1,3^

The present case report describes the history of a sea lion keeper, where the sea lions were infected with tuberculosis and transmitted the disease to the keeper. This is also a warning for the possible transmission of this disease in settings other than health care, which can also be considered an occupational disease.

## Case report

We report the case of an otherwise healthy 27-year-old white male zookeeper, without any work-related conditions. Relevant personal history information included childhood viral meningitis and smoking habits (five pack-years). He denied any other relevant history or long-term medication use. He has worked as a zookeeper caring for sea lions and dolphins for about 10 years, without direct contact with other animals. His daily tasks include directly feeding the animals, which involves washing and cutting the fish. Despite the presence of veterinarians, the zookeepers, due to greater proximity and familiarity with the animals, also perform blood collections when necessary. In addition, he also cleans the spaces where sea lions and dolphins live, washes the animals, and uses positive reinforcement training techniques with them.

The zookeeper sought permanent medical care (PMC) at a private hospital for a 3-week history of dry cough, fever (maximum temperature: 38.5ºC), sweating, and unquantified weight loss. He denied sputum production, asthenia, or adynamia. He tried acetaminophen and ibuprofen without improvement. He reported an epidemiological context of contact with sea lions and dolphins, of which he takes care, with tuberculosis in the last 4 months. A screening chest radiograph performed at the Respiratory Diagnostic Center (RDC) (1 month before this appointment) showed no abnormalities; he was asymptomatic at that time. Laboratory analysis revealed a slight increase in inflammatory parameters (C-reactive protein, 6 mg/dL), but sputum was negative for acid-alcohol resistant bacilli (AARB). It should be noted that AARB testing at an early stage of the disease is often negative. He was discharged from the PMC with a referral for bronchoscopy approximately 4 days later in a public hospital.

Bronchial lavage, however, was positive for AARB. Molecular drug resistance testing showed no resistance to isoniazid or rifampicin. Lavage cultures were positive for the *M. tuberculosis* complex.

**Figure 1:**
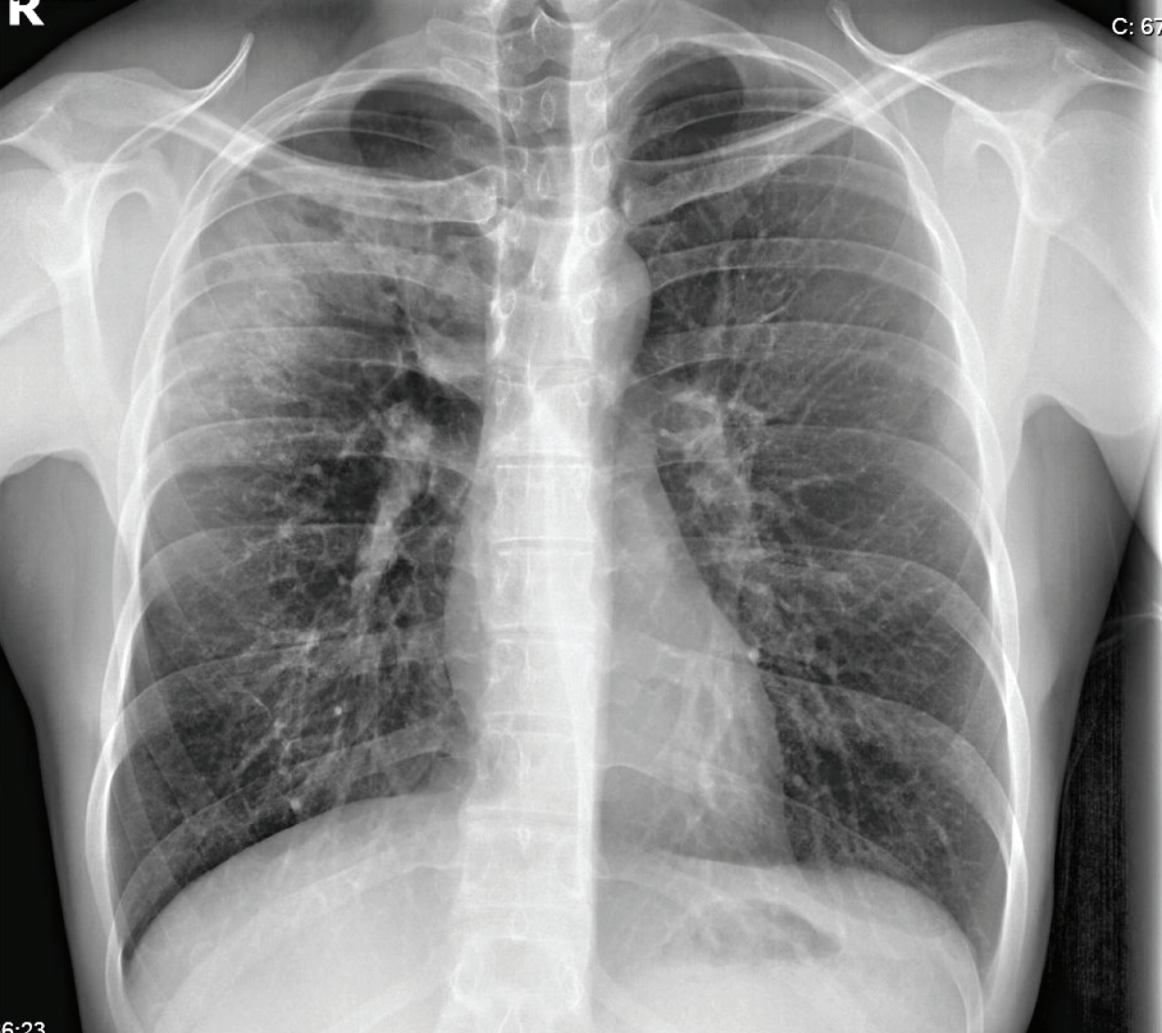
Posteroanterior chest radiograph.

**Figure 2:**
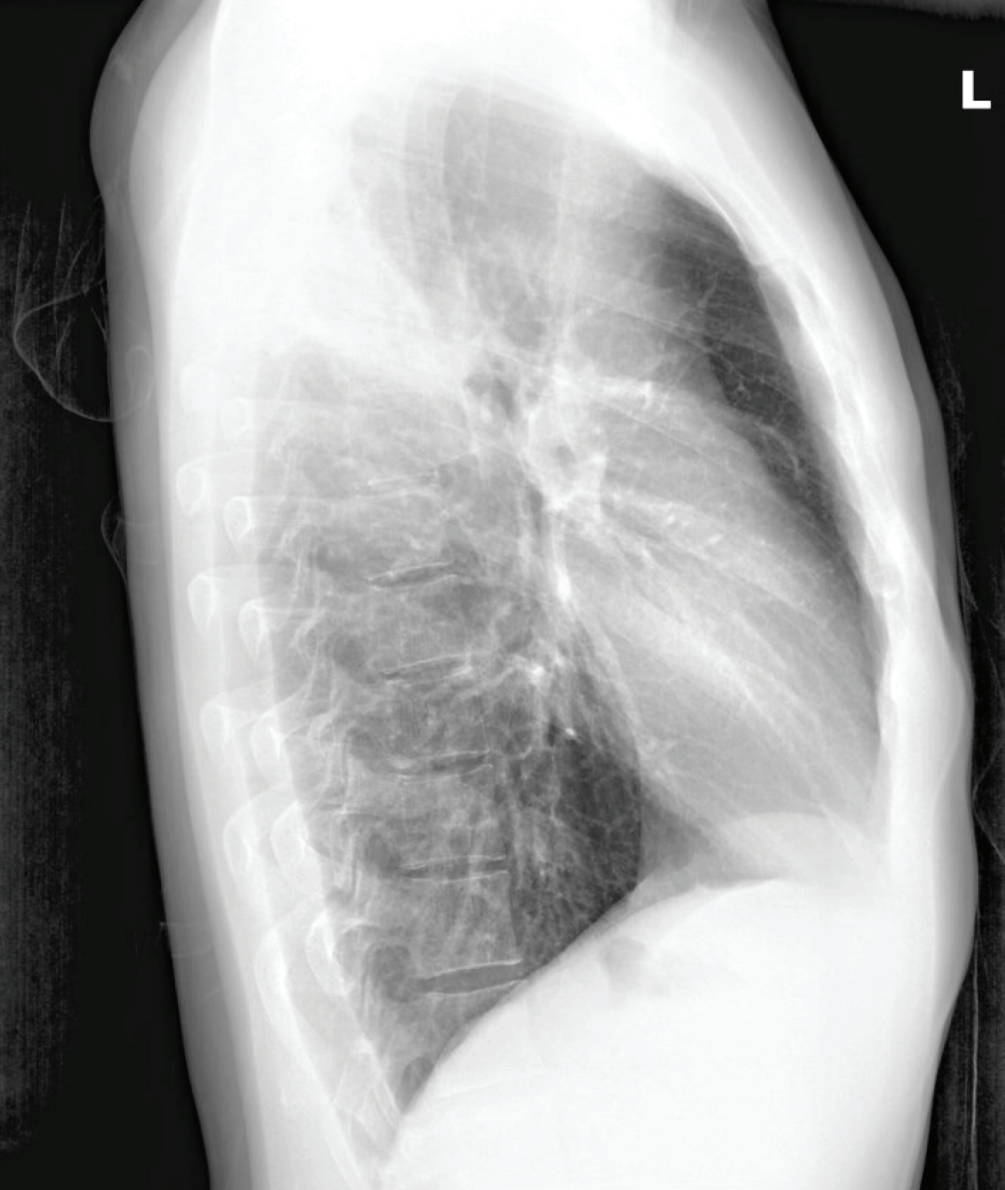
Lateral chest radiograph.

On physical examination, he was eupneic at rest, with peripheral oxygen saturation of 97% on room air, heart rate of 110 beats per minute, sustained and symmetrical breath sounds on lung auscultation, mild crackles in the right upper third, and no palpable adenopathies at the submandibular, cervical, axillary, or supraclavicular level. He had negative serology for human immunodeficiency virus, hepatitis C virus, and hepatitis B virus, with mild monocytosis, without anemia. A chest radiograph showed evidence of hypotransparency in the right upper third with central cavitation, without pneumothorax or pleural effusion (Figures 1 and 2). The patient was referred to the RDC, where treatment was initiated with antituberculosis drugs. The patient still reports some episodes of dry cough and right chest pain, worsened by coughing.

During treatment, the patient was on sick leave for about 10 months. Treatment under direct observation was given at the local health center.

Subsequently, he was referred to an occupational physician and monitored by regular follow-up visits after completion of treatment. Given the diagnosis of tuberculosis in an occupational setting, we proceeded to the mandatory reporting of occupational disease.^4-6^ Zoo animals diagnosed with tuberculosis, of which the patient was the caretaker, were isolated and properly treated and monitored by the veterinary team, having also been subject to pharmacological treatment, thus not limiting his subsequent professional activity. All other professionals who in any way were in contact with the animals were duly screened and monitored at the RDC. Soon after the diagnosis of the animals, measures were taken, such as mandatory face mask use by all professionals who came into contact with these animals and restricted access of people to them.

Regarding occupational health, the patient is fit for work and has resumed his job as a zookeeper.

## DISCUSSION

Tuberculosis remains an important public health problem worldwide, being considered one of the most serious infectious diseases and causing major family, social, and economic dysfunctions.^7^ The incidence in Portugal has shown a decreasing trend over the last 3 decades, although it is still high compared with that of other countries of the European Union.^8^ Data for 2019 reveal an incidence of about 23.2 new cases per 100,000 population in Europe. However, it should be noted that 29 countries of the European Union have a rate of 9.2 cases per 100,000 population, indicating that tuberculosis is far from being evenly distributed across the European region. Approximately 83% of estimated cases occur in 18 countries, where the incidence is five times higher than the European Union average. As for Portugal, in 2019, the incidence was 17.2 new cases per 100,000 population, below the European rate but still much higher than that of most countries of the European Union.^8-11^

*M. bovis*, together with *M. tuberculosis, M. africanum, M. caprae, M. canettiie*, and *M. microti*, form the *M. tuberculosis* complex and are responsible for most cases of tuberculosis in humans and animals.^9^

In the occupational setting, tuberculosis continues to be a matter of great concern and attention, most often associated with the hospital environment or occurring among health care professionals in contact with infected patients. However, the case reported here shows another, less usual occupational setting in which this type of contact can also occur. Recurrent infection can affect persons who deal directly with infected animals, such as animal caretakers, veterinarians, butchers, and farm workers, among others.

Animal tuberculosis is an infectious disease of chronic evolution responsible for a small but relevant percentage of cases of tuberculosis in humans, with an extensive host range. Transmission by infected animals occurs mainly through exhaled air, respiratory secretions, and feces (due not only to intestinal lesions but also to the swallowing of contaminated mucus originating from the airways). Other products may also be contaminated, such as milk and dairy products, urine, vaginal and uterine discharges, and purulent material from skin abscesses or from open lesions in peripheral lymph nodes.^9,10^

In active pulmonary tuberculosis, the patient may have only mild symptoms, such as malaise accompanied by anorexia, fatigue, and weight loss, which develop gradually over several weeks, or more specific symptoms. Cough is undoubtedly the most common symptom. Hemoptysis usually occurs only with cavitary tuberculosis (due to granulomatous damage to vessels, or because of fungal growth in the cavity). Low-grade fever is common but not invariable. Night sweats are a classic symptom, but they are not specific only to tuberculosis. As for the onset of dyspnea, it may result from involvement of the lung parenchyma, spontaneous pneumothorax, or pleural tuberculosis with effusion.^1,7,8^

The infected worker should be monitored regularly for possible lung function abnormalities or residual lesions, which, in any case, are rare findings in patients with tuberculosis diagnosed early and treated properly, without complications. In this occupational setting, it is also crucial to monitor and treat all animals diagnosed with tuberculosis, as well as all people present in this community. Close contacts of both humans and animals with pulmonary tuberculosis should undergo tuberculosis screening, which consists of clinical examination, chest radiography, tuberculin test, and/or interferon gamma release assay (IGRA). The diagnosis of tuberculosis as an active disease is based on laboratory identification of bacteria in the *M. tuberculosis* complex in organic products (sputum in most cases). Confirmation of the disease and susceptibility profile to antituberculosis drugs allows the choice of correct treatment.^1,9^

It is therefore important to implement programs such as One Health, promoted by the World Health Organization (WHO), which aims to design and implement projects, policies, legislation, and research in which multiple sectors communicate and work together to achieve better public health outcomes. Multiple professionals with a range of expertise working in different sectors, such as public health, animal health, plant health, and the environment, should join forces to support One Health approaches by detecting, responding to, and preventing outbreaks of zoonoses and food safety issues.^12^

Finally, it is expected that the warning generated by this case can be used by occupational health teams, namely those who are responsible for periodic surveillance of workers’ health in the animal sector.
